# Brain age gap estimation using attention-based ResNet method for Alzheimer’s disease detection

**DOI:** 10.1186/s40708-024-00230-1

**Published:** 2024-06-04

**Authors:** Atefe Aghaei, Mohsen Ebrahimi Moghaddam, Alzheimer’s Disease Neuroimaging Initiative

**Affiliations:** Faculty of Computer Science and Engineering, Shahid Beheshti University, Tehran, Iran

**Keywords:** Alzheimer’s disease, Attention, Brain age gap, Structural MRI, 3D-Resnet

## Abstract

This study investigates the correlation between brain age and chronological age in healthy individuals using brain MRI images, aiming to identify potential biomarkers for neurodegenerative diseases like Alzheimer's. To achieve this, a novel attention-based ResNet method, 3D-Attention-Resent-SVR, is proposed to accurately estimate brain age and distinguish between Cognitively Normal (CN) and Alzheimer’s disease (AD) individuals by computing the brain age gap (BAG). Unlike conventional methods, which often rely on single datasets, our approach addresses potential biases by employing four datasets for training and testing. The results, based on a combined dataset from four public sources comprising 3844 data points, demonstrate the model's efficacy with a mean absolute error (MAE) of 2.05 for brain age gap estimation. Moreover, the model's generalizability is showcased by training on three datasets and testing on a separate one, yielding a remarkable MAE of 2.4. Furthermore, leveraging BAG as the sole biomarker, our method achieves an accuracy of 92% and an AUC of 0.87 in Alzheimer's disease detection on the ADNI dataset. These findings underscore the potential of our approach in assisting with early detection and disease monitoring, emphasizing the strong correlation between BAG and AD.

## Introduction

The human brain experiences ongoing changes throughout the lifetime, which are a normal aspect of the aging process and do not necessarily indicate any pathological conditions [1]. As individuals age, various alterations manifest in the brain. The observed changes encompass a reduction in the overall quantity of gray matter, enlargement of the ventricles, compromised integrity of the white matter resulting from factors such as myelin sheath damage, a decrease in the number of cells involved in neurotransmission, and a reorganization of brain function. Furthermore, it is important to note that neurodegenerative diseases, such as dementia, have the potential to impact the structural integrity of the brain and expedite the aging process. Consequently, acquiring a more comprehensive understanding and representation of typical brain aging can facilitate the differentiation between normal aging and neurodegenerative processes. Therefore, a better understanding and modeling of the natural aging of the brain can help to disentangle these two processes and improve the diagnosis of neurodegeneration in the early stages. Within the field of neuroscience, there is an emerging inclination to utilize brain MRI scans for the purpose of constructing age prediction models [2–4]. In this case, the difference between the brain age and the chronological age, Brain Age Gap (BAG) is a highly reliable and heritable biomarker that reflects pathological processes in the brain.

The estimation of brain age can be achieved through the utilization of both traditional and machine learning methodologies. This can be accomplished by considering the entire brain, specific regions of interest, or patches [5–7]. In a study conducted by researchers [8], a novel approach utilizing T1-weighted MRI images was introduced. This approach aimed to predict brain age by analyzing metrics at both the region and voxel levels. Machine learning algorithms, including regression-based algorithms, have also been employed for the purpose of predicting brain age. In a previous study [9], conventional machine learning techniques such as kernel regression were utilized to forecast brain age based on whole-brain MRI images of individuals spanning different age groups, including children and adults. In a separate study [10], various machine learning algorithms, such as Support Vector Regression and Binary Decision Tree, were evaluated to determine their efficacy in predicting brain age. Researchers have conducted investigations on multiple regression algorithms, including Relevance Vector Regression and Twin Support Vector Regression, in order to estimate brain age using various imaging modalities [5, 11, 12]. In another study, the Gaussian Process Regression algorithm is utilized for brain age estimation and mortality as well [13]. While traditional machine learning algorithms have demonstrated satisfactory outcomes in specific domains of medical image analysis, such as brain age estimation, they encounter difficulties related to manual feature extraction. On the other hand, deep learning methods possess the ability to automatically extract features, thereby allowing them to effectively handle unstructured data and solve intricate problems. In the subsequent paragraph, we will examine methodologies rooted in deep learning that are utilized for the estimation of brain age.

The development of deep learning methods for brain age estimation using T1-weighted structural MRI images has been undertaken by some researchers like [14–16]. A study conducted by researchers [17] has emphasized that conventional machine learning algorithms, such as Lasso regression and Support Vector Regression, are insufficient in capturing the intricate relationships present within brain structure. Consequently, the utilization of deep learning models becomes imperative. In [7], a Convolutional Neural Network (CNN) was used to make predictions on brain age using both raw and pre-processed MRI data. A separate study [18] introduced a three-dimensional convolutional neural network (3D CNN) model for estimating brain age. The study showcased the model's exceptional performance in comparison to 2D CNN models. In a previous study [3], a Fully Convolutional Network (SFCN) was proposed for brain age estimation. The SFCN was built upon the VGG Net model and utilized a comprehensive dataset. The results obtained from this approach were highly impressive. In addition to convolutional neural network (CNN) models, attention-based methodologies, such as transformers, have been successfully employed for the purpose of estimating brain age. These models utilize their attention mechanisms to attain exceptional performance. In [19], researchers proposed a graph transformer that utilizes regions of interest. Additionally, another study [20] introduced a global–local transformer, both of which demonstrated remarkable outcomes.

Deep learning models have been shown to improve decision-making performance, however, it is important to note that they typically require a significant amount of data in order to achieve optimal results. The issue of data leakage poses a substantial challenge within the field of medical image processing. In order to tackle this challenge, researchers have proposed the utilization of transfer learning approaches. These approaches involve fine-tuning pre-trained models such as ResNet, which is a highly advanced method in the field of classification [21, 22]. The application of transfer learning has also been extended to the estimation and prediction of brain age. A deep transfer learning model was proposed for brain age estimation using MRI data in reference [23]. In a separate study [24], a ResNet-based transformer was proposed for the purpose of brain age estimation and Alzheimer's disease classification.

The investigation of progressive disorders has led to increased interest in brain age estimation methods. These methods are focused on the early diagnosis of Alzheimer's disease and predicting the conversion from mild cognitive impairment to Alzheimer's disease [24]. As mentioned before, one well-established biomarker for evaluating brain pathology is the brain age gap. This refers to the disparity between an individual's estimated brain age, as determined by a model, and their actual chronological age [25]. Consequently, there have been several studies in the scientific literature focused on detecting Alzheimer's disease using brain age as a diagnostic indicator. In [26], the concept of brain age was proposed as an index for Alzheimer's disease, utilizing MRI imaging techniques. The research conducted aimed to create a comprehensive automated framework for the estimation of brain age in both healthy individuals and those diagnosed with mild cognitive impairment and Alzheimer's disease. This framework utilized MRI scans as the primary data source. An assessment was conducted to examine the correlation between brain age and anatomical MRI measurements, including gray matter volume, white matter volume, cerebrospinal fluid volume, cortical thickness, and hippocampal volume. The findings revealed a clear correlation between brain age and conventional Alzheimer's screening tools, as well as anatomical MRI measurements. In study [27], the brain age of patients with Alzheimer's disease and patients with Parkinson's disease was assessed and compared. support vector regression (SVR) is used on T1-weighted MRI images, along with measurements of gray and white matter volumes. The study revealed notable variations in average age-related disparities in gray matter (9.29 ± 6.43) and white matter (8.85 ± 6.62) among individuals diagnosed with Alzheimer's disease.

Given that the majority of the aforementioned methods are classification-based, necessitating labeled MRI images for each age group, there is a constraint on the estimation to the age groups encompassed in the training set. Consequently, the models will exhibit a bias towards the particular training set employed. In addition, another issue that causes model bias is the use of one dataset as training dataset and test dataset. using several datasets as training datasets instead of one dataset makes the model not biased on one of them and increases its generalizability. Since the main purpose of proposing the method in the articles is to be able to use it in real data, if the model is biased on that dataset, it usually does not give a good answer. Additionally, existing literature demonstrates that attention-based models are currently considered state-of-the-art techniques in brain age estimation [19, 20]. The comparison of the introduced methos is shown in Table 1. Some of these methods use traditional Machine Learning approaches. In these methods, features are extracted handcrafted and then a regression algorithm is applied to brain age estimation from these features. The deep learning methods dived on three main approaches: Deep Neural Network, CNN, and Transformer. For Deep Neural Networks which did not use CNN, features are still extracted manually and then fed into a deep neural network. Since CNN extract features automatically, using CNN may increase performance. One of the CNN methods is Residual Convolutional Neural Network (ResNet). A ResNet architecture has a feed-forward signal which skips the CNN block and directly affects the outputs. In this paper, instead of simple CNN, we used Resnet, which is a combination of residual blocks. Also, transformers have performed very well in image processing due to their attention-based nature, however, they require a lot of data to get good results. We took advantage of this nature of attenuation and increased efficiency by adding attenuation layers between residual blocks. If we had more data, probably the use of transformer methods would have given us better results.

**Table 1:** The comparison of the related works

References	Modality	Dataset	Brain working Region	Method	Error
[8]	T1	IXI, OASIS	Region and voxel level	Regression	MAE = 5.75 4.97
[9]	T1	NIH MRI	Whole Brain	Conventional ML	MAE = 1.1
[10]	T1	IXI, OASIS	Gray Matter (GM), White Matter (WM), cerebrospinal fluid (CSF)	Quadratic SVR Decision tree	MAE = 4.63 to 7.14
[5]	T1	IXI	Whole brain	relevance vector machine (RVM)	MAE = 5
[11]	T1	Custom dataset (954 MRI)	Voxel Level	post-hoc linear regression	MAE = 5.1
[12]	T1 (IXI)	IXI	GM, WM, CSF	Improved twin SVR	MAE = 2.69
[13]	T1	LBC1936	GM, WM	Gaussian process	MAE = 5.02
[15]	T1	Cam-CAN	Whole Brain	CNN	MAE = 4.06
[16]	T1	MGHBCH NIH-PD, ABIDE, BeijingEN, IXI, DLBS, OASIS	Whole Brain	CNN + Transformer	MAE = 2.38
[16]	T1	ABIDE, BeijingEN, IXI, ICBM, ADNI	Patch-based	Deep neural network	MAE = 4.7
[7]	T1	BANC	GM, WM	CNN	MAE = 4.16
[18]	T1	Custom (over 1000 MRI)	Voxel-Level	3DCNN	MAE = 3.67
[3]	T1	UK Biobank, PAC 2019	Whole Brain, GM, WM	Simple fully convolutional network (SFCN)	MAE = 2.9
[20]	T1	BGSP, OASIS, NIH-PD, ABIDE, IXI, DLBS, CMI, CoRR	Patch-based	Global–local transformer	MAE = 2.7
[20]	Multi-Modal MRI	UK Biobank	ROI-based	Graph-transformet	MAE = 2.71
[23]	T1 Blood Parameteres	ADNI, IXI, OASIS	GM	Deep transfer learning	MAE = 3.96
[26]	T1	IXI, OASIS	GM, WM, CSF	SVR	MAE = 4.02
[27]	T1	IXI, ADNI, OASIS, PPMI	GM, WM	SVR	MAE = 4.38

The contributions of the paper are as follows:

- We have designed a new model which is a combination of 3D Residual blocks, Attention blocks, and linear regression to accurately forecast brain age.
- In order to optimize performance, we have integrated our deep learning approach with SVR, a conventional machine learning method.
- In order to enhance the generalizability and address concerns related to data leakage, we have integrated multiple datasets into our training set.

Additionally, we conducted an evaluation of the estimated brain age as a sole biomarker for Alzheimer's disease classification, yielding favorable outcomes.

The subsequent sections of the paper are structured as follows: In Sect. 2, a comprehensive explanation of our proposed method is presented. Section 3 showcases the experimental findings, while Sect.4 offers an extensive analysis and discussion of the research paper.

## Method

In this section, we explore the methodology and datasets employed. Section 2.1 introduces the datasets used for training and testing the proposed model. Subsequently, the proposed method is discussed across four sub-sections. Section 2.2 offers an overview of the proposed method, while Sect. 2.3 explains the image pre-processing steps taken to prepare the data for the model. Section2.4 delves into a detailed explanation of the proposed network, which combines Regression-based 3D-Attention-ResNet and SVR. Finally, Sect. 2.5 outlines the process of Alzheimer’s disease classification using the estimated brain ages.

### Datasets

In this paper, four famous datasets, which are ADNI (Alzheimer’s Disease Neuroimaging Initiative) [28], Autism Brain Image Training Data (ABID) [29], Brain Genomics Support Project (BGSP) [30], and Cambridge Centre for Ageing and Neuroscience (cam-CAN) [30] are used. A combination of these four datasets is used as a training and test set for evaluation of the proposed method. The details of these datasets are shown in Table 2. A brief explanation of each of these datasets is as follows.

**Table 2:** The demographic information of the introduced datasets

Dataset	Number of data	Number of train data	Number of test data	Age range	Age average
ADNI	998	748	250	50–90	77.2 ± 5
BGSP-Harvard	1571	1179	392	19–30	21.5 ± 2.8
ABIDE	621	466	155	7–64	16.8 ± 7.5
Cam-CAN	654	490	164	18–90	54.3 ± 18.5
total	3844	2883	961	7–90	42.4

#### ADNI

The Alzheimer's Disease Neuroimaging Initiative (ADNI) is a research study focused on the diagnosis, prediction, and detection of progression in individuals with Alzheimer's disease. The datasets encompass four distinct collections, namely ADNI-1, ADNI-2, ADNI-Go, and ADNI-3, which have been systematically gathered since 2004. This study incorporates various biomarkers, including MRI, PET, fMRI, as well as genetic and biological data, collected at regular intervals of approximately six months. These biomarkers are obtained from individuals classified as Cognitively Normal (CN), Mild Cognitive Impairment (MCI), and Alzheimer's disease (AD). This paper utilizes a sample of 203 healthy subjects from the study to train the proposed brain age model. Due to the availability of multiple MRI images captured at different time points for each subject, the total count of MRI volumes has now reached 998.

#### GSP-harvard

The Brain Genomics Support Project (BGSP) is a comprehensive collection of neuroimaging, behaviour, cognitive, and personality data. This large-scale imaging dataset includes information on over 1,500 healthy subjects, encompassing variables such as age, BMI, and gender. The dataset comprises a single high-resolution structured MRI image along with several resting state fMRI scans. This study utilizes structured MRI images in conjunction with the subjects' gender.

#### ABIDE

The Autism Brain Imaging Data Exchange (ABIDE), which comprises ABIDE1 and ABIDE2, was released in 2012. Each dataset contains data collected from over 24 laboratories. The dataset comprises functional magnetic resonance imaging (fMRI) and structural magnetic resonance imaging (sMRI) data from a total of 539 individuals diagnosed with autism spectrum disorder (ASD) and 573 control participants classified as cognitively normal. The age range of the subjects spans from 7 to 64 years, with an average age of 14.7 years. The dataset utilized in this study comprises data obtained from control subjects.

#### Cam-CAN

The Cambridge Centre for Ageing and Neuroscience (Cam-CAN) was established in October 2010. The Cam-CAN project comprises a cohort of approximately 3000 individuals ranging in age from 18 to 90 years. Within this cohort, there are approximately 700 individuals who have undergone structural Magnetic Resonance Imaging (sMRI), magnetoencephalography (MEG), functional Magnetic Resonance Imaging (fMRI) both during resting state and task-based conditions, as well as completed cognitive experiments.

### An overview of the proposed method

**Fig. 1:**
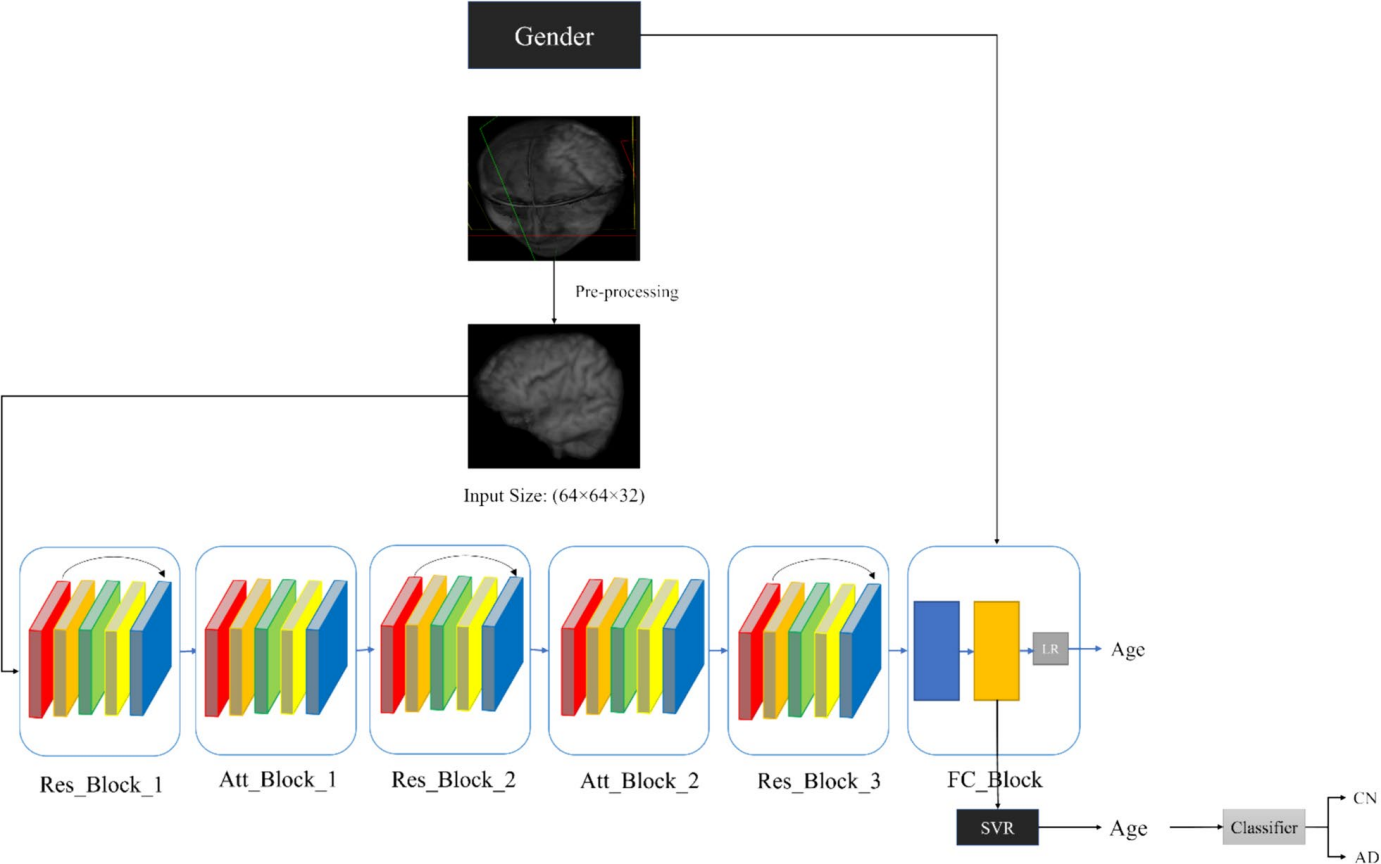
An overview of the proposed method

As depicted in the Fig. 1, the first step of the proposed method is pre-processing, which is described in Sect. 2.3 in details. In the second step, the pre-processed 3D volumes are inputted into the deep learning model, which consists of three Res Blocks and two Attention Blocks. In this article, we have used the combination of two mechanisms used in the ResNet [31] and self-attenuation [32]. When the network becomes deep, the gradients of the loss function during the backpropagation become extremely small, and this causes patterns not to be learned in the final layers. Residual blocks help prevent this by adding an input layer to some convolution layer during the network. Also, attention layers help model learns the relationship between parts of the input data to better decision making. The features extracted from the proposed 3D_Att_ResNet model are combined with the patient's gender as metadata. These combined features are then passed into the fully connected block. The features extracted from our proposed deep neural network are utilized as input for the Support Vector Regression (SVR) model, which is employed to estimate the brain age. This step is explained in Sect. 2.4. To Alzheime’s disease detection, the model is tested on both healthy individuals and those diagnosed with Alzheimer's disease. The proposed method is utilized to estimate the brain age of these subjects. The proposed method demonstrates its performance by utilizing the brain age gap, which is the discrepancy between chronological age and estimated brain age, as the only biomarker for classifying Alzheimer’s disease. Further details regarding the classification of Alzheimer’s disease can be found in Sects. 2.5.

### Pre-processing

**Fig. 2:**
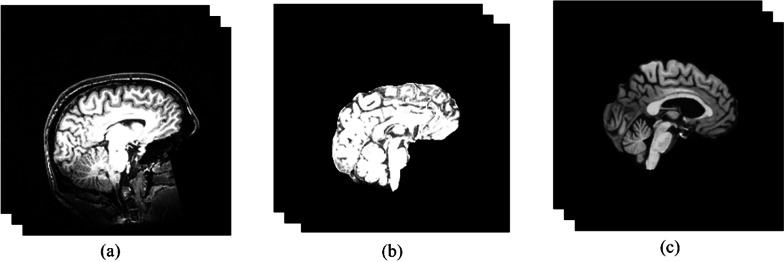
preprocessing of the images. The results on the sagittal side of one image from ABIDE dataset. (To show the results better, we show some slices of the 3D volume). **a**; the original image, **b**: after brain extraction using the brain mask, **c**: After N4 bias field correction

For brain age estimation using sMRI, it is imperative to extract the brain from MRI images. Hence, the brain is extracted from the scalp and skull utilizing DeepBrain tools, which is a customized U-Net model. The brain extraction outcome is depicted in Fig. 2b. Also, Intensity Non-Uniformity (INU) is a significant artifact observed in brain MRI images. To mitigate this artifact, the N4 bias field correction method [33] is employed for image processing. The outcomes of the bias correction are depicted in Fig. 2.c. Furthermore, it is imperative to ensure that the size of the images used in this paper's training and test datasets are appropriately aligned, given that multiple datasets are employed. In order to achieve this objective, the MRI volumes are resized to a specified dimension. The size of ADNI, ABIDE, Cam-CAN, and BGSP-Harvard datasets are ADNI: $256 \times 256 \times 256$, $176 \times 256 \times 256$, $256 \times 256 \times 192$ and $192 \times 192 \times 144$ respectively, they are resized to $64 \times 64 \times 32$.

### Proposed model

This section provides a comprehensive description of the proposed method. Regarding to Fig. 1, the proposed model is a fusion of Support Vector Regression (SVR) and the 3D_Attention_ResNet regression model. Appendix 1 shows the details of architecture blocks of Fig. 1 consists of number of 3D convolution layers, the size of the output feature maps, and the parameters of each layer.

The architecture comprises an Input Layer responsible for receiving the input data. It is followed by three Resnet and two Attention blocks. Each Resnet block comprises of 3D convolutional layers, Batch Normalization, residual layer, a shortcut between the Input Layer (x) and the last BN Layer (F(x)), ELU activation function, and Max-pooling. The shortcut is represented by a pointwise sum of the output feature map from these two layers, as denoted by Eq. 1.

1 $$H(x) = CNN(x) + F(x)$$

Each of the blocks in the architecture includes the following steps: extraction the feature maps using 3D convolutional layers, applying an activation function to introduce non-linearity, normalizing the activations using batch normalization to enhance training stability, and down-sampling through max-pooling to reduce complexity. Also, the proposed model consists of two Attention blocks. Each block includes 3D convolutional layers, Batch Normalization, ELU activation function with alpha equal to 1.0, Attention layer, and Max-pooling. The structure of each block is shown in Eq. 2, 3, and 4.

2 $$\begin{gathered} Q = query\_embedding = 3DCNN \hfill \\ V = value\_embedding = 3DCNN \hfill \\ att = Attention(Q,V) \hfill \\ \end{gathered}$$

3 $$\begin{gathered} Con = Q \oplus att \hfill \\ BN = BatchNorm(Con) \hfill \\ \hfill \\ \end{gathered}$$

4 $$Output_{i} = Max\_Pooling(ELU(BN))$$

where *Q* (Query embedding) and *V*(value embedding) are a 3DCNN layer. An attention layer is calculated using Q and V according to Eq. 3. After that, attention layer and Q are concatenated and fed into a Batch Normalization layer. Finally, ELU activation function is applied to the output of the Batch Normalization layer and fed into max-pooling to reduce the dimension. If the number of attention block is* i*, the output of the *ith* attention block is obtained by Eq. 4.

The proposed model concludes with a Fully Connected Block, which comprises a Flatten layer, a Dense layer, an ELU activation function, and a Dropout layer. First, the output is flattened to transform it into a one-dimensional vector. Next, the flattened output is passed through a fully connected dense layer. An ELU activation function, and Dropout is applied to the model. Additionally, in the proposed model, the gender of the subject is utilized as metadata. A one-dimensional array is added to the model as gender of the subjects. It is concatenated to the image feature map. The two feature maps were combined and utilized in a linear regression model to estimate brain age. Once the training model has been completed, the feature maps extracted from the model are fed into the SVR model to estimate the final brain age based on Eq. 5, where F_l_ is the feature map of the last of the proposed method and G is Gender of the subject. Alg.1 depicts the process of predicting brain age using proposed method.

5 $$y = SVR(F_{l} \oplus G)$$

### Alzheimer’s disease detection

As previously stated in the introduction, the disparity in age between the estimated age determined by analyzing brain MRI images and the actual chronological age is a significant biomarker in the diagnosis of Alzheimer's disease. Hence, the brain age gap serves as the only biomarker utilized for the classification of Alzheimer's disease. We employ two class classifiers to classify individuals with Alzheimer's disease and individuals without any cognitive impairments. In order to achieve improved outcomes, classifiers are employed for the purpose of classification. The utilized classifiers consist of Logistic regression [34], Support Vector Machine (SVM) [35], which is widely recognized, as well as two Ensemble learning models: Adaboost [36], and XGBoost [37]. In order to achieve improved outcomes, an Ensemble learning approach is employed to combine the results of these three classifiers through major voting. We show the AD detection using the proposed method and BAG in Alg.2.

**Algorithm 1:**
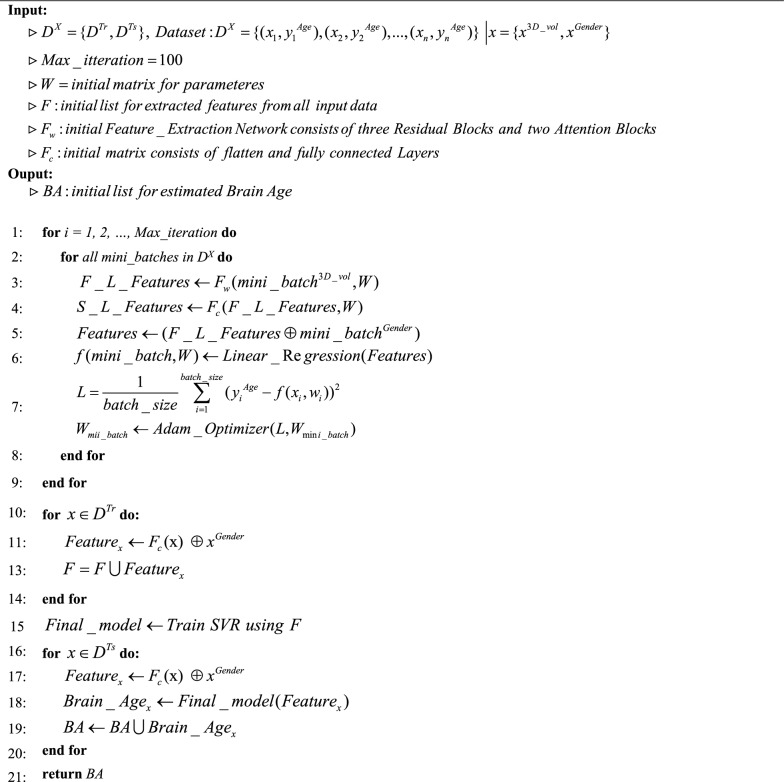
Brain age prediction using attention based 3DResNet and SVR

**Algorithm 2:**
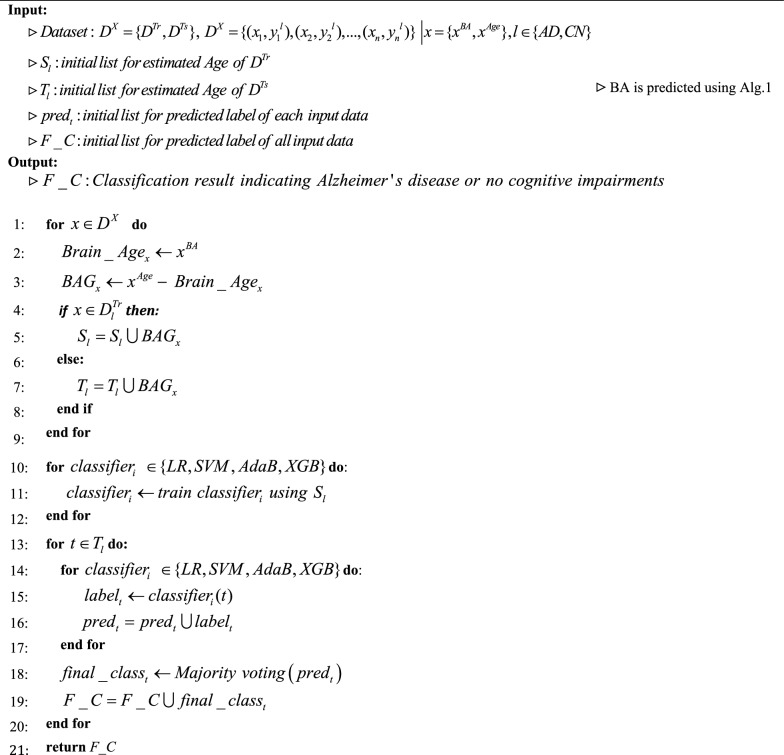
Alzheimer’s disease detection using brain age gap

## Results

### Implementation details

The proposed model for brain age estimation comprises three Residual Blocks and two Attention Blocks. Each Res Block consists of two 3D convolution layers with a kernel size of 3 × 3 × 3, Batch Normalization, Elu activation function with an alpha value of 1, one 3D convolution layer with a kernel size of 1 × 1 × 1, a concatenation layer, and Maxpooling with a size of 2 × 2 × 2 and stride 2. The number of features in the first, second, and third Res Blocks are eight, 32, and 128, respectively. Each Attention Block is composed of three 3D convolution layers with a kernel size of 3 × 3 × 3, Batch Normalization, Elu activation function with an alpha value of 1, an attention layer, and Maxpooling with a size of 2 × 2 × 2 and a stride of 2. Additionally, the number of features for the first and second Attention Blocks are 16 and 64, respectively. At the conclusion of the network architecture, there is a flattening layer, followed by a Dense layer with a size of 128 and a l2 regularization term. Additionally, there is a dropout layer with a rate of 0.2, an input layer for capturing the gender of the subjects, and a concatenation layer for combining the features of MRI images with the gender information of the subjects. The Adam optimization algorithm is employed with a learning rate of 0.0007, in conjunction with the Mean Squared Error loss function. The convergence is achieved after 100 epochs. The network has been implemented utilizing the GPU Tesla T4 and Intel(R) Xeon(R) CPU @ 2.20 GHz within the Keras library in Python 3.9.

### Analysis of results obtained from the proposed method

**Fig. 3:**
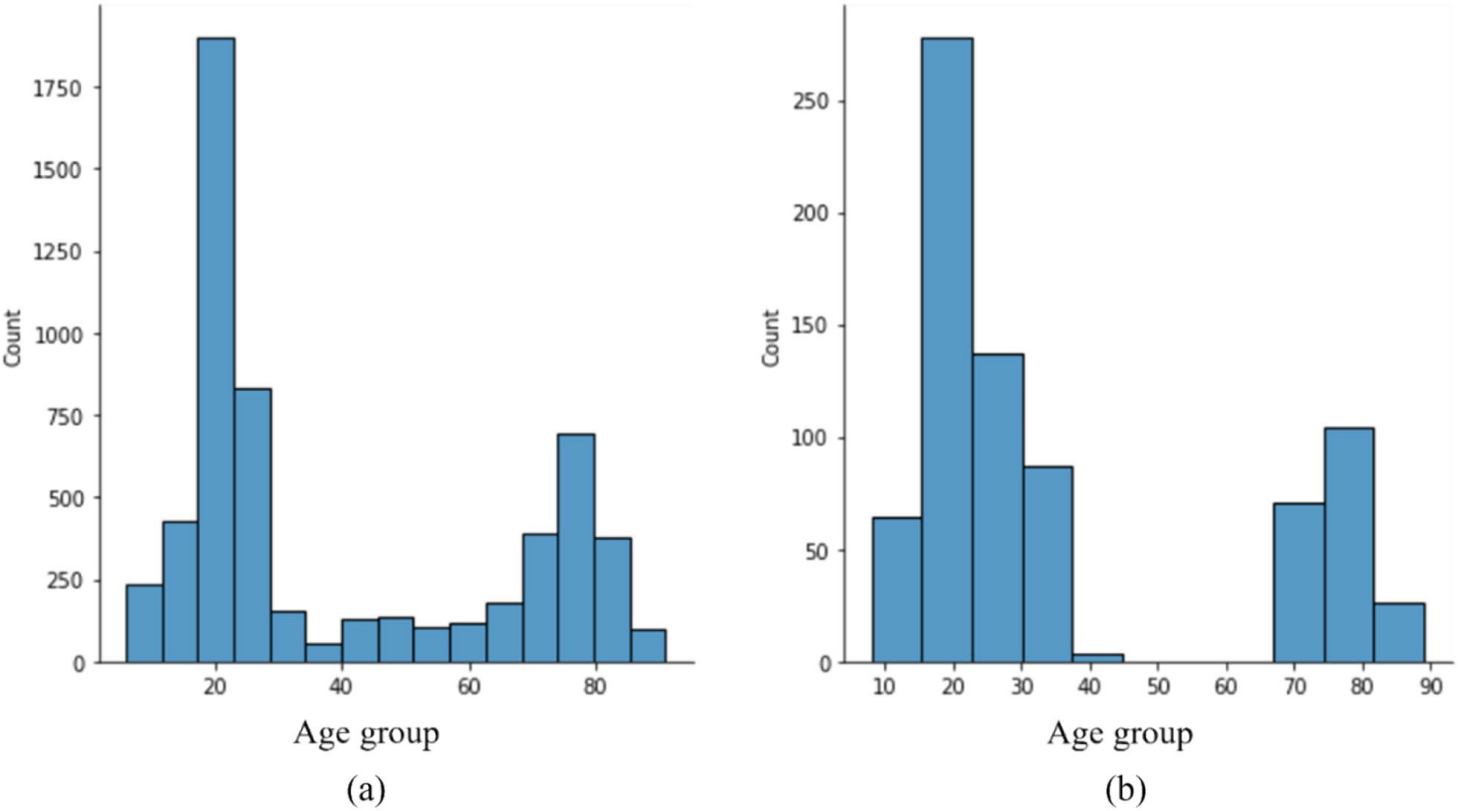
the distribution of the chronological age of the combination of the introduced datasets. The horizontal axis is the age group and the vertical axis is the count of each age group **a**: the distribution of the chronological age of 2883 training data and **b** the distribution of the chronological age of 961 test data

In this section, the proposed method is evaluated using a combination of the four datasets that have been previously introduced. We used subject leave out approach to evaluate our proposed method. The datasets that have been introduced are consolidated with 25 percent of the subjects designated as the test set. The remaining data is allocated for the training and validation sets. The number of training and test data are illustrated in Table 2. Also, the distribution of the training and test sets can be observed in Figs. 3a, b, respectively. The proposed model is subjected to training and validation processes utilizing specific datasets. After the model has achieved convergence, it is subsequently evaluated using an independent test set. The Support Vector Regression (SVR) is utilized on the feature map of the last layer, yielding the ultimate results.

**Fig. 4:**
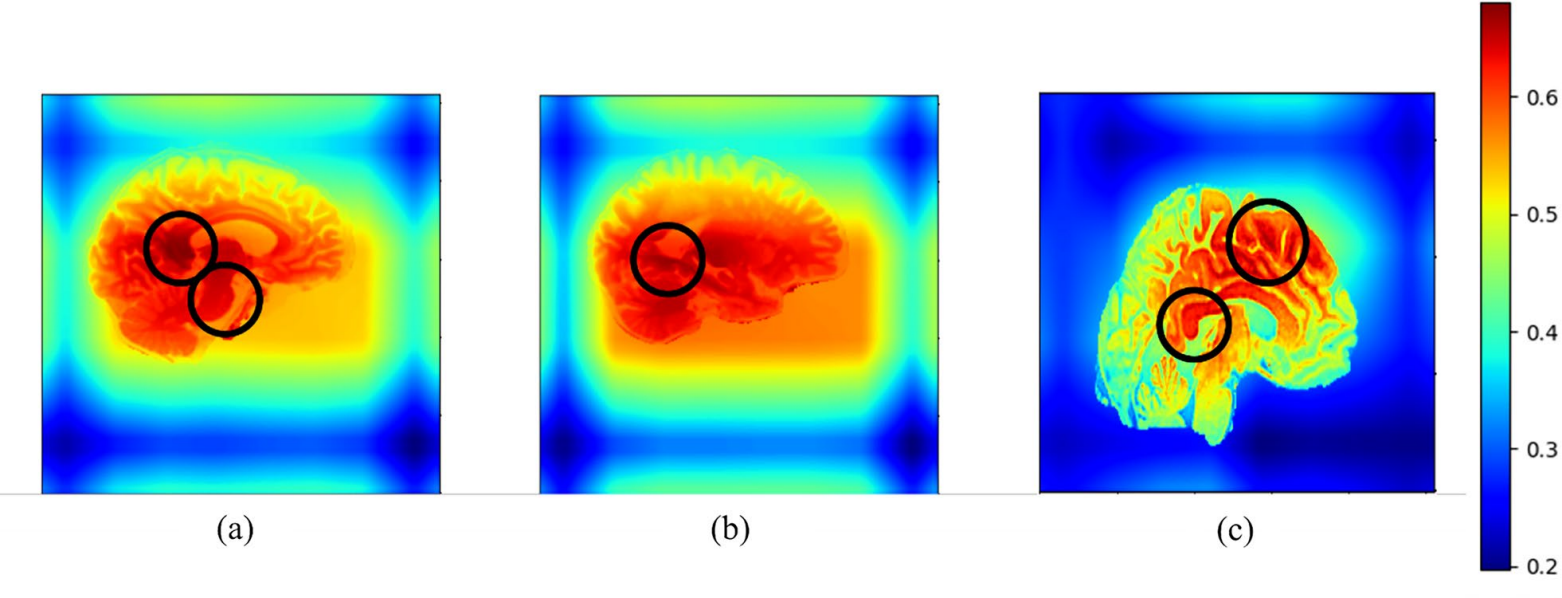
The Heatmap (The most important parts of the brain which are involved in brain age estimation in our proposed method) of the brain of three different subjects, which the age of subject **a** and **b** are more than 60 and subject **c** is between 30 and 40 years old. The most important parts for brain age estimation are marked with a circle which are ventricles, hippocampus, and some parts of parietal lobe

As explained in the introduction section, with aging, some changes in the brain including a decrease in the total amount of gray matter, enlargement of the ventricles [38, 39], changes in the white matter, including damage to the myelin sheath, a decrease in the number of cells involved in neurotransmission and reorganization of the brain can be seen [40]. Also, at younger ages, age detection is done through other parts, including the parietal lobe [20]. Figure 4 is the heatmap obtained from our proposed model using Grad-CAM algorithm [41]. The parts of the brain that were more important in our model for age estimation are shown in dark red and we have circled them. As shown in the figure, the proposed model for ages over 60 years Fig. 4(a) and (b) made the diagnosis based on the temporal lobe including the hippocampus and ventricles, and for ages younger than 40 years Fig. 4(c) the parietal lobe sections as well. Is taken into consideration.

#### Ablation study

In this section, we have added a complete ablation study. We have used some other methods instead of ResNet blocks and the results are shown in Table 3 In the first row, the model is 3D-Resnet and Attention layers have been removed. In the third and fourth rows, we replace 3D-Resnet with 3D-CNN, in the fourth row the attention layers are added and the method is the combination of 3D-CNN and one attention layers. In the last row, we replace the whole network with transformer. It is worth noting that although transformer is one of the best models for image processing, in this work, since there is not enough data to train transformer, 3D-Resnet-Attention has better results. Based on the results of the models in Table 3, it can be seen that 3D-Resnet-Attention has achieved the best results compared with the other models on our dataset. According to these results, we choose this model and combine it with a conventional model (SVR), the results show the performance of the model is better when we use SVR for brain age estimation.

**Table 3:** The comparison of the proposed method with some other models

Row	Model	MAE	PCC	SRCC	CI
1	3D-resnet	2.53	0.989	0.96	[1.71,3.67]
2	3D-Resnet-Attention	2.13	0.993	0.961	[0.13,0.47]
3	3D CNN	3.7	0.98	0.89	[1.16,1.66]
4	Attention-3D CNN	4.7	0.97	0.84	[-0.66,0.14]
5	Transformer	3.6	0.97	0.88	[1.21,1.80]
6	3D-resnet-Attention + SVR (proposed)	2.05	0.993	0.961	[0.03,0.37]

**Fig. 5:**
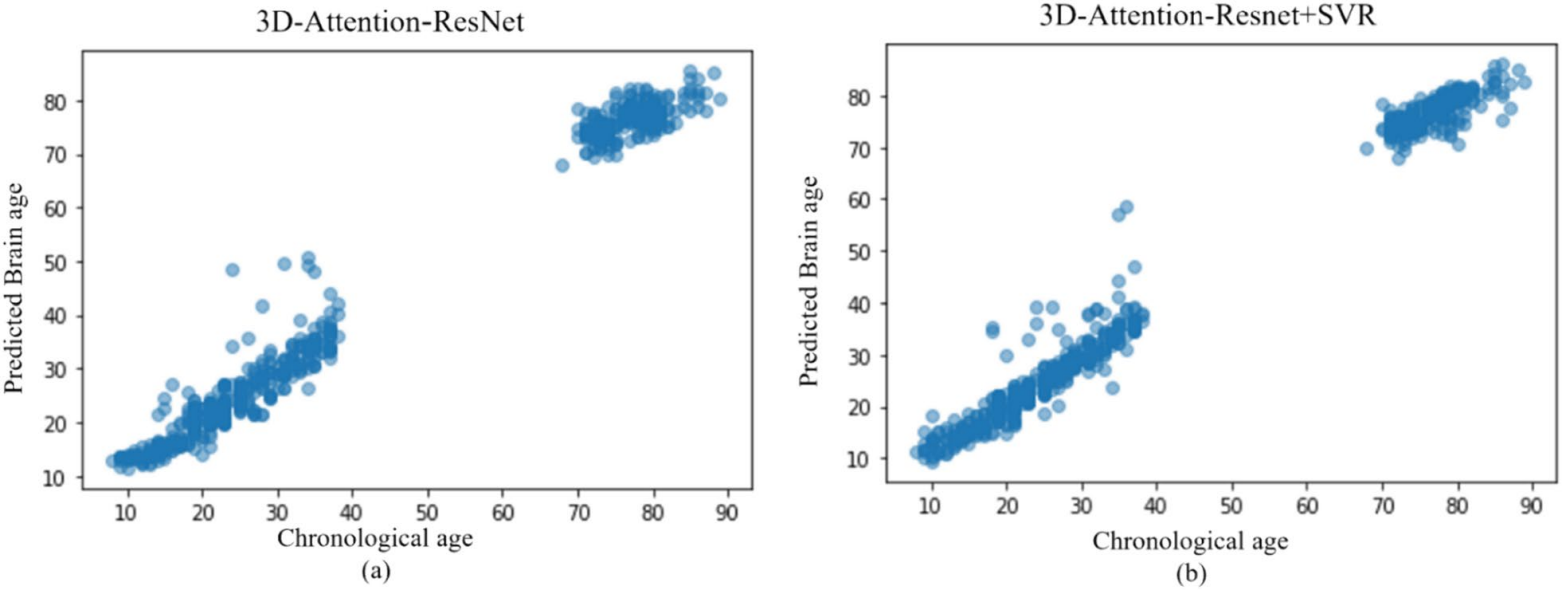
the scatter plot of the chronological age and the brain age estimated using the proposed method on 961 test data. **a** the results without the SVR and **b** the results using the SVR model

Furthermore, the scatter plot of the relationship between the chronological age and the predicted brain age for the proposed model is demonstrated in Fig. 5a, b depict the scatter plot both with and without Support Vector Regression (SVR). The data presented in the Figure demonstrates that the proposed model accurately predicts the age of the majority of healthy subjects in the test dataset. Furthermore, the comparison of the two Figures demonstrates that the utilization of Support Vector Regression (SVR) leads to an enhancement in performance.

We have changed the number of attention layers from 1 to three layers, and have shifted attention layer from second to third and fourth to fifth layer, the results are shown in Table 4. The results show that two Attention layers in Second and fourth layers have obtained better results.

**Table 4:** Comparison the results of different numbers of attention layers

#Attention Layers	Shift Attention layer	Validation MAE
1 layer	–	4.38
2 layers	–	3.71
3 layers	–	5.28
2 layers	✔	5.27
3 layers	✔	6.21

We compare some learning rates from 0.01to 0.00001 in Table 5. Based on the results obtained from validation set which are shown in the second column of Table 5, the best Learning Rate is 0.0007.

**Table 5:** The comparison of the results of the different Learning Rates for optimization function on validation data

Learning Rate	Validation MAE
0.00001	8.65
0.0001	3.73
0.001	4.17
0.01	10.82
0.0007	3.71

**Fig. 6:**
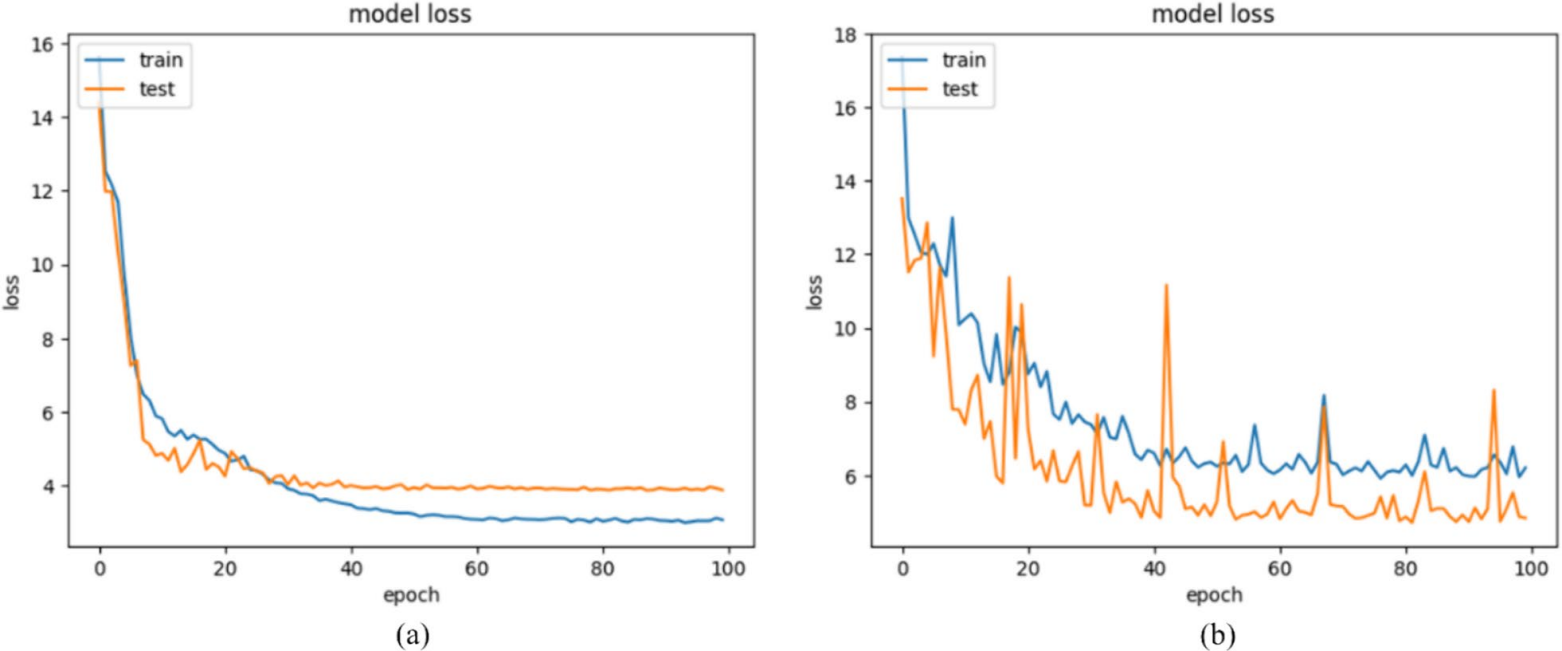
Comparison of FLU and ReLU activation function. **a**: ELU, **b**: ReLU

Moreover, to select the best activation function for hidden layers, we train our model using both ELU and ReLU activation function. We show the train and validation loss per epoch activation function in Fig. 6. The Figure shows that the proposed model converged better using ELU activation function. Also, we test the model obtained from both ELU and ReLU activation function and the results are demonstrated in Table 6. The results show that ELU activation function outperforms ReLU.

**Table 6:** Comparison the results of ReLU and ELU activation function in hidden layers

Method	Activation Function	MAE
3D-Attention-ResNet-	ReLU	3.73
3D-Attention-ResNet	ELU	2.05

Finally, to understand the effect of the pre-processing (extracting brain from skull and scalp and N4 bias correction) removed this part and results have been compared in Table 7. As Table 7 shows using the pre-processing is needed to improve results.

**Table 7:** Comparison the results of the proposed method with and without pre-processing

Method	Pre-processing	MAE	PCC	RMSE
3D-attention-resnet	✔	2.05	0.99	3.44
3D-attention-resnet	–	9.68	0.67	12.3

### Alzheimer’s detection

In this paper, the proposed method is utilized to predict the brain age of cognitively normal (CN) and Alzheimer's disease (AD) subjects from the ADNI dataset. The dataset used in this section is the combination of test data of brain age estimation (for CN subjects) and a random subset of AD subjects. In other words, 25% of CN subjects of ADNI dataset (250 MRI images) which are used for brain age estimation test, together with 250 randomly selected MRI images of AD subjects are utilized for Alzheimer’s disease classification. This is significant as the brain age gap, which refers to the disparity between brain age and chronological age, serves as a valuable biomarker for the detection of Alzheimer's disease. The data is presented in Table 8, which provides a comprehensive overview of the details.

**Table 8:** Demographic information of cognitively normal and Alzheimer’s disease subjects from the ADNI dataset

Group	Number of scans	Number of training scans	Number of test scans	Age range	Average age	Male %	Female %
CN	250	212	38	62–88	75 ± 7.2	51	49
AD	250	212	38	55–93	74.8 ± 7.5	45	55

**Fig. 7:**
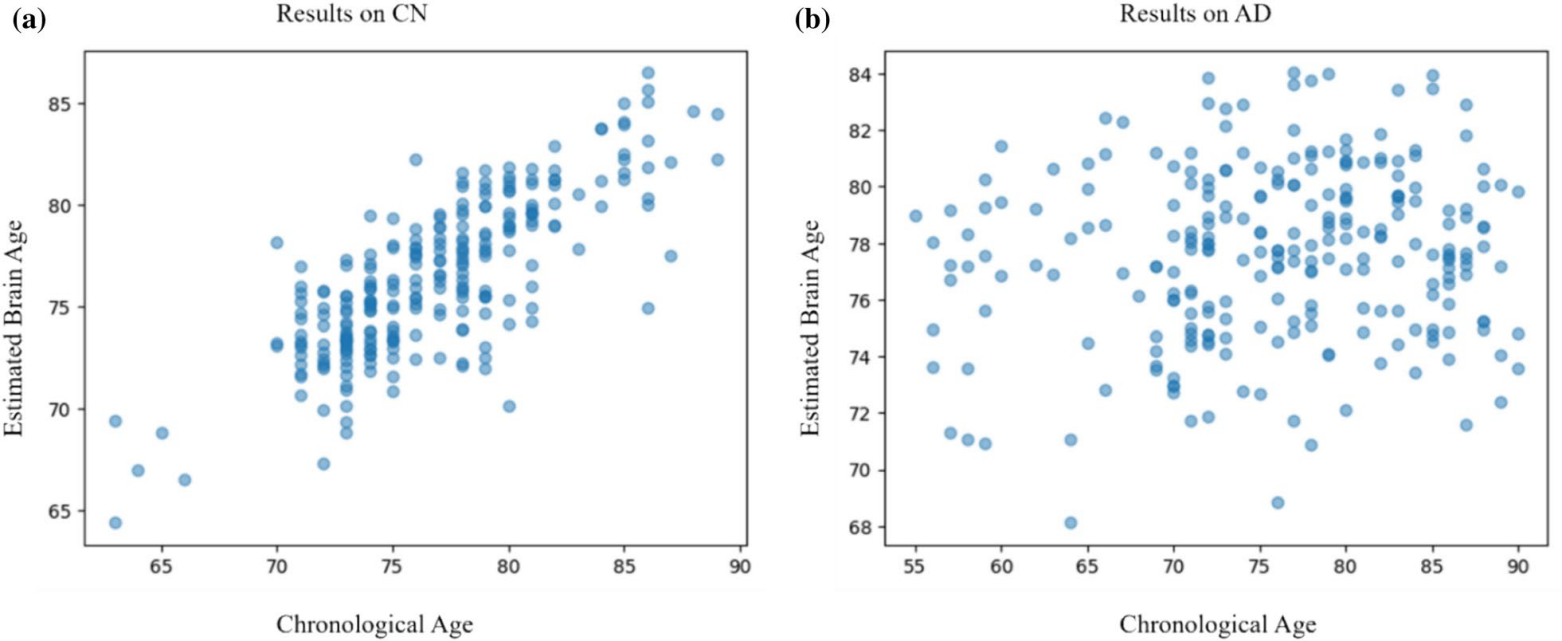
the scatter plot of the chronological age and the brain age estimated using the proposed method on 500 test data of ADNI dataset. **a** the results on Cognitively 250 Normal subjects and **b** the results on 250 Alzheimer’s disease patients

**Fig. 8:**
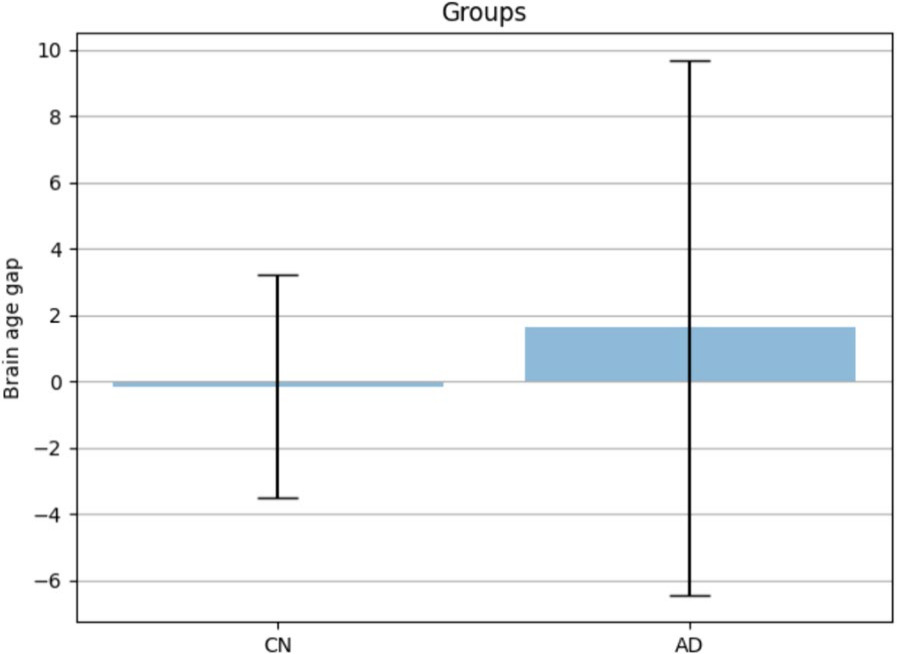
Brain Age Gap of the 250 Cognitively Normal (CN) and 250 Alzheimer’s disease (AD) subjects

The estimated brain age gap between the AD and CN groups is determined by calculating the difference between the predicted brain age, using the proposed method, and the chronological age of the subjects. Figure 7a, b depict the scatter plots illustrating the relationship between the chronological age and the predicted brain age of the CN and AD subjects, respectively. The disparity in brain age between subjects with normal cognitive function (CN) and those with Alzheimer's disease (AD) is similar. The disparity in brain age between the two groups is illustrated in Fig. 8. The observed brain age gap among CN subjects is 2.6 ± 1.8, while the brain age gap among AD subjects is 6.5 ± 2.8.

The brain age gap that was acquired was inputted into four binary classifiers, namely Logistic Regression, SVM, AdaBoost, and XGBoost. We consider 15% of the data (76 scans) as test and the others are applied to the model as training set. In order to enhance the outcomes, the combination of these models is acquired through majority voting. The performance of the proposed method is assessed using Precision, Recall, F1 score, and accuracy, as defined in Eqs. 6, 7, 8, respectively.

6 $$Precision = \frac{TP}{{TP + FP}}$$

7 $${{Recall}} = \frac{TP}{{TP + FN}}$$

8 $$F1 = 2 \times \frac{{Precision \times {Recall}}}{{Precision + {Recall}}}$$

9 $$Accuracy = \frac{TPR + TNR}{2} = \frac{TP + TN}{{TP + FN + TN + FP}}$$

**Fig. 9:**
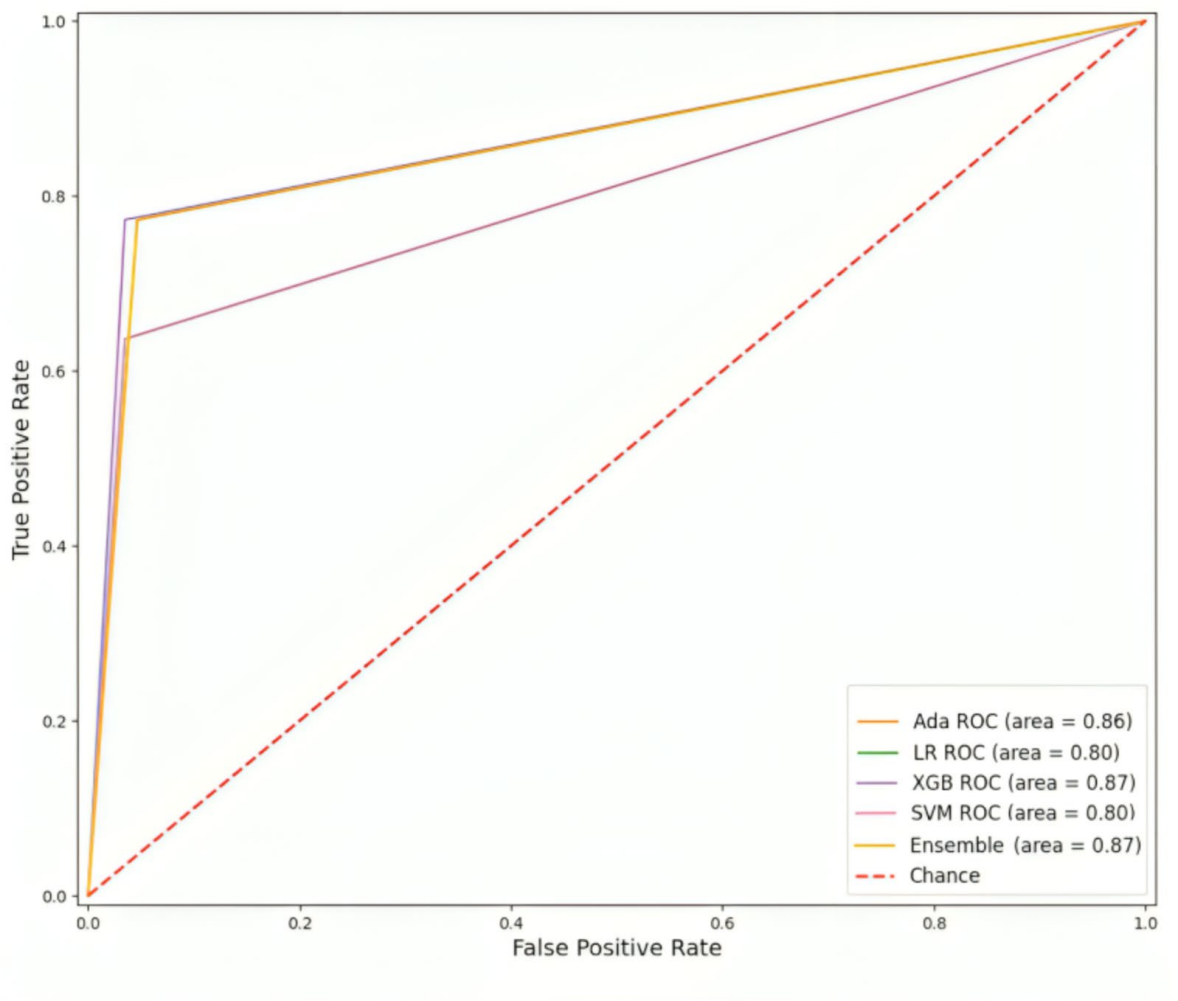
The ROC curve of Alzheimer’s disease classification using Logistic Regression, Adaboost, XGboost, SVM and Ensemble learning

The values of these criteria are presented in Table 9. According to the Table, it is evident that Adaboost and XGBoost outperformed the SVM and Logistic Regression. Additionally, the combination of classifier results through major voting, known as Ensemble learning, yielded the most favorable outcome, which are mentioned with bold values in Table 9. Based on the obtained results, the method demonstrates an accuracy rate of 92%. Moreover, the ROC curve of Alzheimer’s disease classification is illustrated in Fig. 9. As shown in the Figure, the Area Under the Curve (AUC) of the ROC curve is 0.87. The results indicates that the considered biomarker exhibits strong performance in effectively classifying subjects with Alzheimer's disease (AD) from those without cognitive impairment (CN).

**Table 9:** The average of Accuracy, Precision, Recall, and F1 score of the proposed method on 76 test data 38 CN and 38 AD)

Model	Precision	Recall	F1 score	Accuracy
SVM	0.75	0.81	0.78	0.90
Logistic regression	0.70	0.86	0.77	0.89
AdaBoost	0.80	0.77	0.79	0.91
XGBoost	0.85	0.77	0.80	0.92
Ensemble	**0.85**	**0.76**	**0.81**	**0.92**

### Comparison with state-of-the-art

To show the performance of our proposed method, we compared the results of the proposed methods with the results of some state-of-the-art methods in Table 10. We have compared our results with other state-of-the-art methods using some metrics such as r (Pearson Correlation Coefficient), R^2^ (Coefficient of Determination), RMSE, and MAE. As we have already stated, one of the advantages of our method is that due to the use of several different datasets with different distributions as training set, the generalizability of the model to new data is high. This claim can be seen in the first, fifth and seventh rows of the Table. We separate Cam-CAN dataset once and use the rest of the datasets for training the model, and then we test the model on Cam-CAN dataset the results are in the first row of the Table. Comparing the results with the method introduced in [15, 42], which used a 3D CNN, and a combination of CNN and MLP respectively, shows that our model has a much better performance. Also, we separate the ABIDE dataset once and perform the training with the rest of the datasets and then teste on ABIDE dataset, and compare with the method proposed in [43] as shown in fifth and sixth rows on Table 10. The results show that although the MAE of this method is slightly better, our method has also achieved a very good results and the difference of MAE rate between the two methods is very small and insignificant. Also, to show that the model works well even with a small amount of data, we have trained and tested the model once on the IXI dataset and compared it with the results of the method that calculated brain age by using XG Boost classification method [44], which these two methods have been showed in the fourth and fifth rows. Comparing the results shows that our proposed method has performed much better than that method. In the last row of the Table, the results of the proposed method which is trained and test on whole selected datasets (Harvard, ADNI, Cam-CAN, ABIDE) are shown. The results demonstrated that our proposed method performs well in these data.

**Table 10:** Comparison of the proposed model with the state-of-the-art methods

Row	Method	Train dataset	Test dataset	MAE	RMSE	r	R2
1	Proposed	Harvard, ADNI, ABIDE	CamCAN	2.4	3.46	0.98	0.93
2	3D CNN [15]	nine datasets	CamCAN	4.21	–	0.96	–
3	CNN-MLP [42]	Five datasets	CamCAN	4.91	6.14	–	0.89
4	XGBoost [44]	IXI	IXI	–	15.2	0.45	0.14
5	Proposed	IXI	IXI	8.9	11.87	0.67	0.36
6	Deep NN [43]	ABIDE	ABIDE	2.19	–	0.89	–
7	Proposed	Harvard, ADNI, Cam-CAN	ABIDE	2.73	4.01	0.98	0.93
8	Proposed	Harvard, ADNI, Cam-CAN, ABIDE	Harvard, ADNI, Cam-CAN, ABIDE	2.05	3.17	0.99	0.94

## Discussion

This section comprises two subsections dedicated to the analysis and discussion of the paper. Section 4.1 provides a comprehensive analysis of the significance of brain age estimation, the proposed methodology, and the dataset employed. Additionally, Sect. 4.2 discusses about the utilization of the brain age gap as a potential biomarker for Alzheimer's disease detection.

### Estimation of brain age

As mentioned in the introduction, the aging process induces alterations in several areas of the brain, including grey matter and white matter. The accurate prediction and estimation of brain age through imaging techniques are of crucial significance in diagnosing neurodegenerative diseases. Gaining access to extensive datasets poses a significant challenge, thereby impeding the effective training of models. In order to mitigate potential biases and enhance the robustness of our model, we have integrated four distinct datasets (ADNI, GSP-Harvard, ABIDE, and Cam-can) in this study. This approach allows us to augment the data volume and avoid over-reliance on any single dataset. Attention-based models have shown promising outcomes in various domains. Therefore, we have utilized a 3D ResNet approach that incorporates the attention mechanism. Furthermore, our examination of the available literature has indicated that the Support Vector Regression (SVR) algorithm has demonstrated promising results in the field of brain age estimation. As a result, our methodology integrates two machine learning techniques, namely attention-based ResNet and SVR, in order to accurately estimate brain age. In addition, the results were further enhanced by incorporating gender information of the subjects. The network architecture comprises three 3D ResNet blocks and two 3D attention blocks, which are responsible for extracting feature maps. The feature maps, in addition to the gender information, are subsequently inputted into the Support Vector Regression (SVR) model to acquire the ultimate prediction. The proposed model was evaluated on a test set consisting of 961 samples, which represents 25% of the combined four datasets. The evaluation resulted in Mean Absolute Error (MAE) of 2.05, RMSE of 3.17, Pearson Correlation Coefficient (PCC) of 0.99, R^2^ of 0.94 Spearman’s Rank Correlation Coefficient (SRCC) of 0.96 and 95% Confidence Intervals (CI 0.03–0.37). The obtained results showcase the efficacy of our model in accurately forecasting brain age. Furthermore, a comparative analysis was conducted between our proposed method and some other methods like 3D CNN and transformer. The findings unequivocally demonstrate the superior performance of our approach. In addition, Grad-Cam method is used to show interpretability of the proposed method to detect the most important regions of the brain in determining the age. The results of Grad-Cam demonstrate that decision making of the proposed method is based on the regions which are mentioned in the literature.

Since the distribution of data in each dataset is the same, and if a dataset is used as a training dataset, no matter how big the dataset is, the model will be biased on that dataset and will have less generalizability. One of the advantages of our proposed method is using several datasets as training datasets instead of one dataset that made the model not biased on one dataset and increased its generalizability. Because the main purpose of proposing the method in the articles is to be able to use it in real data, if the model is biased on a dataset, it usually does not give a good answer in real situation. In order to prove this claim, once, we use our biggest dataset (BSGP) as training set and test the model on CamCAN dataset, and also, we separated the Cam-CAN dataset from the training data and trained the model on ADNI, ABIDE, and BSGP datasets, and then we tested it on the Cam-CAN dataset. The results are shown in Table 11. Based on the Table, the MAE of the model in this situation has been 2.4 and if we train the model only on BSGP-Harvard dataset and test in on Cam-CAN dataset, MAE has been 9.8.

**Table 11:** The results obtained from CamCAN dataset using one and the combination of three datasets

Train	Test	MAE
BSGP	CamCAN	9.8
BSGP, ADNI, ABIDE	CamCAN	2.4

Time and required memory of the proposed method is also computed and the results are shown in Table 12 in compare to one of the state-of-the-art methods. Since used hardware are not the same, we have added the details of used hardware as a column.

**Table 12:** Training and Test time and required memory of the proposed method compared to one of the state-of-the-art methods

Model	GPU	Training Time	Test Time (per Image)	Memory
Proposed	NVIDIA Tesla T4 GPU with 12 GB memory	2.06 h	28 ms	8.98 GB
CNN-MLP [42]	Two NVIDIA Titan Xp GPUs with 12 GB memory	5.28 h	Not Reported	Not reported

### Alzheimer's disease detection

The disparity in brain age, which refers to the variance between an individual's chronological age and their estimated brain age, is a dependable biomarker utilized in the identification of neurodegenerative conditions like Alzheimer's disease. To Show the efficiency of BAG for Alzheimer’s Disease detection, we used this biomarker to classify healthy people from Alzheimer's patients. To this aim, first, we selected some data from ADNI as a Cognitively Normal (CN) dataset and an Alzheimer's Disease (AD) dataset. Then, we calculated BAG of each of these data, and finally, the binary classification to classify CN and AD subjects is applied on data. Following that, we utilized four widely recognized classifier algorithms, namely Logistic Regression (LR), Support Vector Machine (SVM), AdaBoost, and XGBoost. We then employed a majority voting approach to combine the outcomes of these algorithms as Ensemble learning. The precision, recall, F1 score, and accuracy metrics for our model were computed as 0.85, 0.76, 0.81, and 0.92, respectively. The results indicate that the proposed method not only estimate age of healthy subjects with high performance but the estimated age can also detect Alzheimer’s disease as well.

## Conclusion and future works

The brain age gap is regarded as an effective biomarker for the diagnosis of Alzheimer's disease due to its direct association with neurodegenerative diseases which are characterized by an unconventional acceleration in brain ageing. This article utilizes a combination of four datasets to enhance generalizability and mitigate potential biases in the model. This article introduces an approach that combines attention-based ResNet with the traditional Support Vector Regression (SVR) method to achieve improved outcomes. By employing this methodology, we have successfully obtained a Mean Absolute Error (MAE) value of 2.05. This result is considered beneficial compared to previous research studies' findings. Interpretability of the proposed method using Grad-Cam is also showed to identify the specific regions of the brain that play a significant role in determining the age of the brain. The results of Grad-Cam demonstrate that the method decides based on the regions that are mentioned in the literature. In order to demonstrate the efficacy of the proposed approach, the BAG has been utilized as the only biomarker for diagnosing Alzheimer's disease, resulting in an accuracy rate of 92% and AUC equal to 0.87. The results indicate that this particular methodology is an appropriate approach for determining brain age. This article utilizes individuals' gender as metadata, while also considering the potential use of other metadata for future works.

One of the limitations of the proposed method is that it is not resistant to noise and artifacts, and noise and artifacts must be removed before entering data into the model. Therefore, in future works, we will try to propose a method that is resistant to noise and artifacts. Also, the proposed method has only been used to classify healthy people from Alzheimer's disease and has not been used to predict Alzheimer's disease, that is, to classify MCI patients. In the future, we will try to check the effectiveness of the model for predicting Alzheimer's disease.

## Data Availability

The authors use ADNI dataset. Data is available in adni.loni.usc.edu.

## Appendix 1

See Table 13

**Table 13:** The details of the proposed method

Layer (TYPE)	Output shape	Param #	Connected to
Input layer	[(None, 64, 64, 32, 1)]	0	[]
CONV3D	(None, 64, 64, 32, 64)	1792	[Input [0][0]ʹ]
CONV3D	(None, 64, 64, 32, 64)	1792	[Input [0][0]ʹ]
Attention	(None, 64, 64, 32, 64)	0	[‘Conv3d [0][0]ʹ, ‘conv3d [0][0]ʹ]
Concatenate	(None, 64, 64, 32, 128)	0	[‘Conv3d [0][0]’, ‘attention [0][0]ʹ
Batch_normalization	(None, 64, 64, 32, 128)	896	[‘Concatenate [0][0]ʹ
ELU	(None, 64, 64, 32, 128)	0	[‘Batch_normalization [0][0]ʹ]
Max_POOLING3D	(None, 32, 32, 16, 128)	0	[‘elu [0][0]ʹ]
CONV3D	(None, 32, 32, 16, 128)	442496	[‘Max_pooling3d [0][0]ʹ]
Batch_normalization	(None, 32, 32, 16, 128)	896	[‘Conv3d [0][0]ʹ]
ELU	(None, 32, 32, 16, 128)	0	['batch_normalization [0][0]'
CONV3D	(None, 32, 32, 16, 128)	442496	['elu [0][0]']
CONV3D	(None, 32, 32, 16, 128)	16512	[‘Max_pooling3d [0][0]ʹ]
Batch_normalization	(None, 32, 32, 16, 128)	896	[‘Conv3d [0][0]’]
Add	(None, 32, 32, 16, 128)	0	[‘Conv3d [0][0]ʹ, Input [0][0]']
ELU	(None, 32, 32, 16, 128)	0	[‘Add [0][0]']
MAX_POOLING3D	(None, 16, 16, 8, 128)	0	[‘elu [0][0]ʹ]
Flatten	(None, 262144)	0	[‘Max_pooling3d [0][0]ʹ]
Fully connected layer	(None, 128)	3355456	['flatten [0][0]ʹ]
ELU	(None, 128)	0	[‘Fullyconnectedlayer [0][0]ʹ]
Dropout	(None, 128)	0	[‘elu [0][0]ʹ]
Gender (Input layer)	(None, 1)	0	[]
Dense	(None, 1)	129	[‘Dropout [0][0]ʹ]
